# Prevalence of Oral Lichen Planus in Patients Referred to the Department of Oral Diseases and Periodontology in Ljubljana, Slovenia 

**DOI:** 10.3290/j.ohpd.c_2665

**Published:** 2026-05-07

**Authors:** Eva Skaleric, Luka Lipar, Anea Mlinar, Nina Hropot Plesko

**Affiliations:** a Eva Skaleric Assistant Professor and Periodontist, Department of Oral Diseases and Periodontology, University Medical Center Ljubljana, and Faculty of Medicine, Ljubljana, Slovenia. Idea, experimental design, literature search, wrote the manuscript.; b Luka Lipar Assistant Professor and Cardiologist, Department of Cardiology, University Medical Center Ljubljana, Slovenia. Statistical analysis, read and approved the submitted version.; c Anea Mlinar Periodontist, Department of Oral Diseases and Periodontology, University Medical Center Ljubljana, Ljubljana, Slovenia. Data extraction, read and approved the submitted version.; d Nina Hropot Plesko Teaching Assistant and Periodontist, Department of Oral Diseases and Periodontology, University Medical Center Ljubljana, and Faculty of Medicine, Ljubljana, Slovenia. Literature search, wrote the manuscript.

**Keywords:** oral lichen planus, prevalence, Slovenia.

## Abstract

**Purpose:**

To assess the prevalence of oral lichen planus and its associated factors among the patients referred to the Department of Oral Diseases and Periodontology in Ljubljana, Slovenia in the period 2022-2023.

**Materials and Methods:**

Out of 1288 patients, 111 patients (8.6%) were diagnosed with oral lichen planus either clinically or by histopathology and/or with direct immunofluorescence assay and included in the study.

**Results:**

79.3% were women and the average age was 63.05 ± 11.94 years. The buccal mucosa was affected most frequently (44.1%) and reticular lesions were the most prevalent (54.1%). Associated skin lesions were found in 5.4% of patients, and 13.5% of the patients also had periodontal disease. 63.1% of the patients reported having at least one systemic disease, 64.0% of the patients reported taking one or more medications. Most of the patients (87.0%) were non-smokers. 60.0% of the patients reported being symptomatic. In 36.0%, the diagnosis was confirmed by histopathology, and in 29.7% of the cases the diagnosis was confirmed by direct immunofluorescence assay.

**Conclusion:**

The results of this study reveal that the prevalence of oral lichen planus in patients referred to the Department of Oral diseases and Periodontology in Ljubljana, Slovenia, is high.

Lichen planus (LP) is a chronic inflammatory mucocutaneous disease that can affect the skin, hair, nails, and mucosal surfaces.^28^ Oral lichen planus (OLP) affects the oral mucosa and is characterized by white lace-like reticular lesions, with or without atrophic or erosive areas.^42^ Six patterns of clinical presentations of OLP are recognized: reticular, atrophic, erosive, papular, plaque, and bullous.^13^ Reticular lesions are the most frequent form of OLP and usually present without symptoms. In patients with atrophic-erosive OLP, burning, soreness, or pain in the oral cavity are present. These symptoms may appear spontaneously or when the patient is chewing.^12^ Mucosal lesions are usually multiple and often have a bilateral distribution. Most commonly, the buccal mucosa, lateral borders of the tongue, and gingiva are affected. Atrophy and ulceration can also affect the gingiva, which is known as desquamative gingivitis. Desquamative gingivitis is seen in approximately 10% of patients with OLP.^18^ Severe desquamative gingivitis can influence the esthetic appearance of patients.^12^

The prevalence of LP worldwide is estimated to be from 0.22% to 5%,^13^ and the incidence of OLP is estimated to be 2.2%.^7^ The incidence is higher in women, smokers, and patients who abuse alcohol.^37^ OLP in children is very rare.^5^

Although several immunological mechanisms of OLP pathogenesis have been proposed, the precise etiology of OLP remains unknown. Frequently, a multifactorial process is considered to be involved, including genetic, psychological, and infectious factors.^25^ Psychological stress, drug intake, and anxiety are known as risk factors.^22^ OLP may be associated with several systemic diseases, such as hypertension, diabetes, graft-vs-host disease, thyroid dysfunction, and hepatitis C virus infection.^16,40^

In cases of classic reticular pattern, the diagnosis can be achieved solely upon clinical examination. When the reticular pattern is absent, an oral biopsy with histopathological examination is required so the suspected diagnosis can be confirmed and dysplasia and cancer can be excluded.^19^ OLP has a low risk of malignant transformation into oral squamous carcinoma, which arises from the squamous cells lining the oral cavity and represents the majority of intra-oral malignant tumors.^2,11,20,34,43^ Its main risk factors are smoking and alcohol consumption, as well as bacteria and microorganisms in the oral cavity.^35^ The mechanism is consistent with the broader model of inflammation-associated carcinogesis.^6,10,17,41,45^

The prevalence of OLP in many parts of the world has been reported, while the information on the epidemiology of OLP in Slovenia is scarce. Only one study by Kovac-Kavcic and Skaleric investigated the prevalence of oral mucosal lesions in 555 randomly chosen Ljubljana citizens, finding a prevalence of 2.7% OLP.^21^

The aim of this study was to investigate the prevalence of OLP, clinical features and other associated factors in patients referred to the Department of Oral Diseases and Periodontology in Ljubljana, Slovenia, in the 2-year period 2022-2023. The hypothesis was that the prevalence of OLP in referred patients is high, as there are no oral medicine specialists in Slovenia and most of the patients with the diseases of the oral mucosa are referred to periodontists and oral surgeons.

## MATERIALS AND METHODS

The study was conducted in accordance with the ethical principles of the Helsinki Declaration (WMA, 2013), and the study protocol was approved by the National Medical Ethics Committee of the Republic of Slovenia (No. 49/08/11). The review protocol was registered and allocated the identification number NCT06787872 in the ClinicalTrials.gov (NIH, U.S. National Library of Medicine).

### Research Subjects

In this study, the data of 1288 patients that were referred to the Department of Oral Diseases and Periodontology during the 2-year period between 2022 and 2023 were analyzed. The following data were obtained from the patients’ diagnostic charts: age, gender, clinical diagnosis with details of clinical presentation, present systemic diseases, medication, and smoking details. One hundred eleven (111) patients (8.6%) with the clinical or histopathological diagnosis of OLP were included in the study.

The inclusion criteria were clinical or histopathological diagnosis of OLP performed using the modified World Health Organization criteria.^39^ Patients who did not fulfill these criteria and patients with suspected lichenoid lesions were excluded from the study.

Demographic and clinical characteristics are summarized with the use of descriptive statistics.

## RESULTS

### Basic Characteristics of the Study Subjects

**Fig 1:**
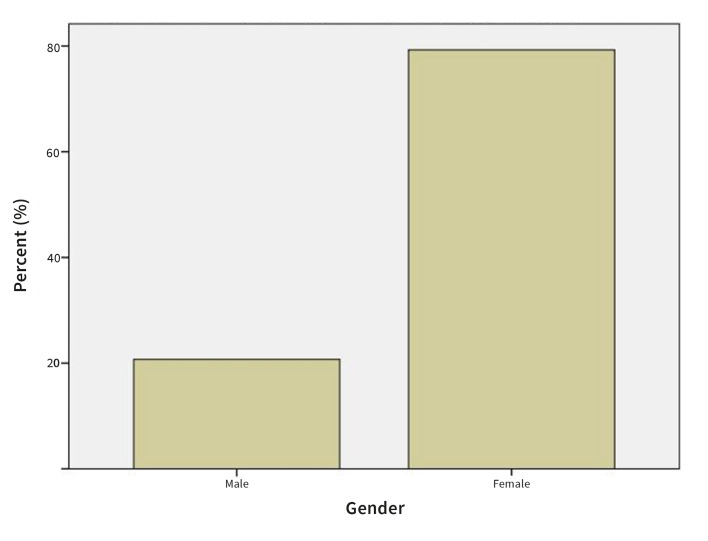
Prevalence of patients OLP by gender.

**Fig 2:**
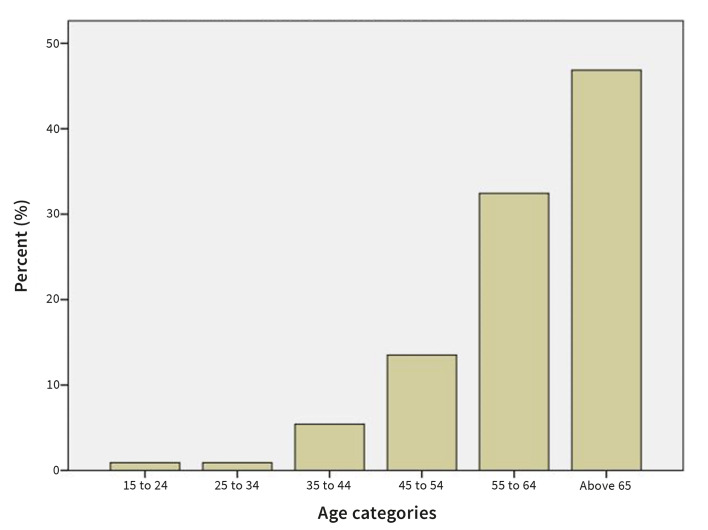
Prevalence of OLP by age.

A total of 111 patients with a clinical or histopathological diagnosis of OLP that were referred to the Department of Oral diseases and Periodontology, University Medical Centre, Ljubljana, Slovenia were included in the study. Among the patients there were 23 (20.7 %) men and 88 (79.3 %) women (Fig 1). The patients were 24 to 86 years old (average 63.05 ± 11.94 years). Prevalence of OLP increased with age from 0.9% in age category 15-24 years to 46.8% in the age category above 65 years (Fig 2).

### Analyses of Related Factors in Patients with OLP

#### Location and type of OLP

**Fig 3:**
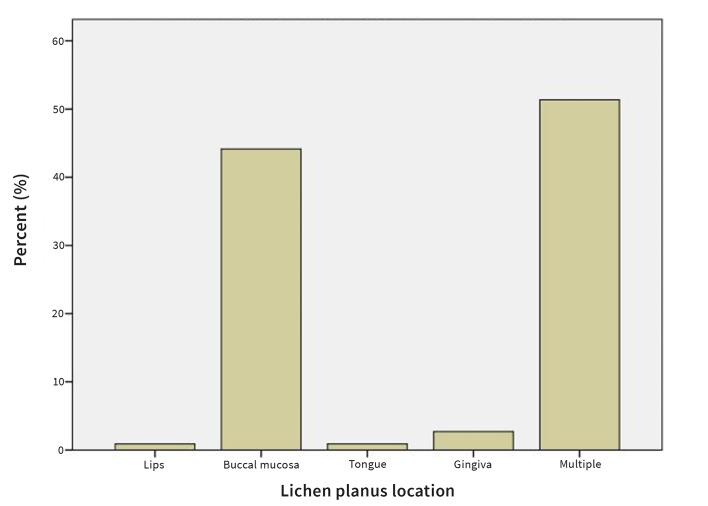
Prevalence of OLP by location.

**Fig 4:**
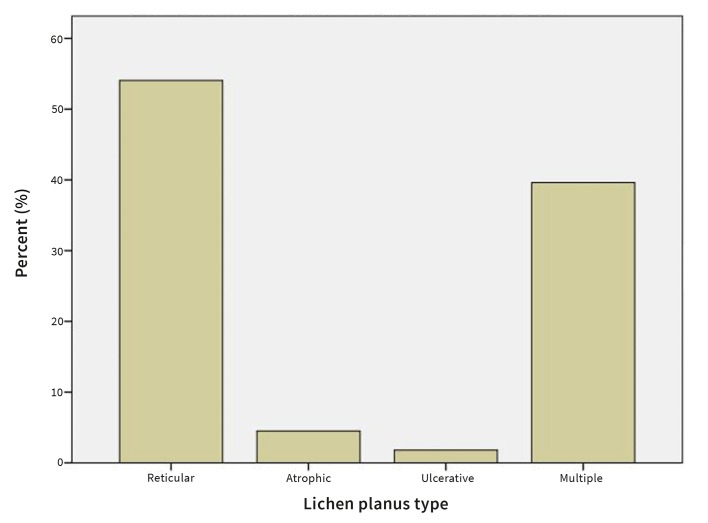
Prevalence of OLP by type.

The buccal mucosa was affected most frequently (44.1%), followed by the gingiva (2.7%). In 51.4%, multiple sites were involved (Fig 3). Reticular OLP was the most prevalent (54.1%), followed by atrophic (4.5%) and ulcerative (1.8%) type. In 39.6% multiple types of OLP were present (Fig 4).

#### Presence of skin lesions and periodontal disease

**Fig 5:**
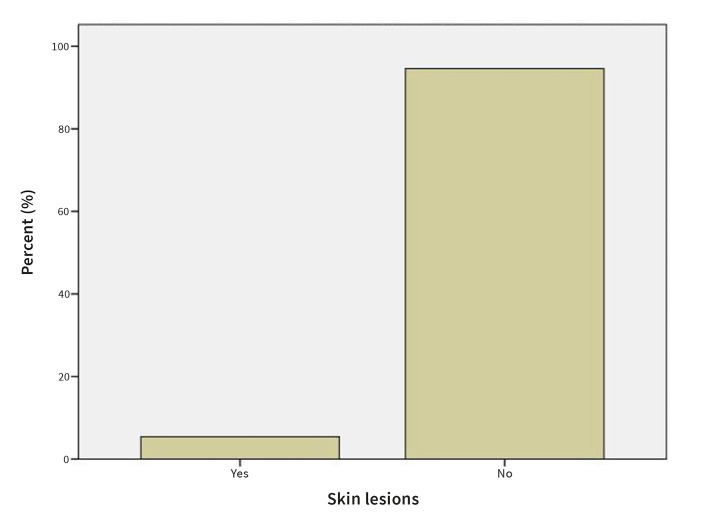
Prevalence of skin lesions in OLP patients.

**Fig 6:**
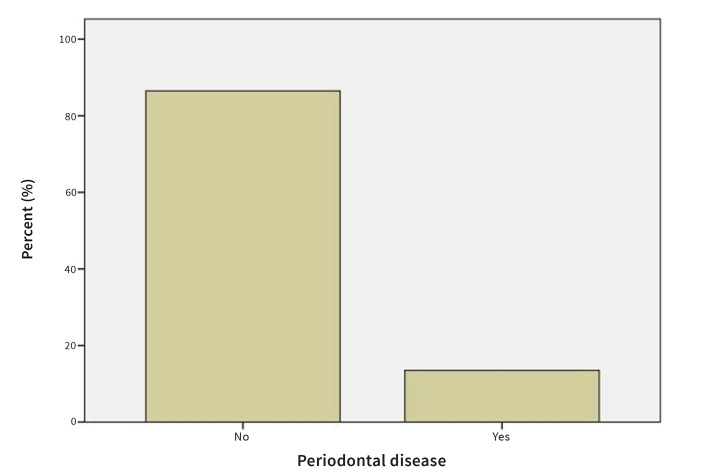
Prevalence of periodontal disease in OLP patients.

In 5.4% of OLP patients, skin lesions were present in addition to oral lesions (Fig 5). 13.5% of patients with OLP also exhibited periodontal disease (Fig 6).

#### Presence of systemic diseases

**Fig 7:**
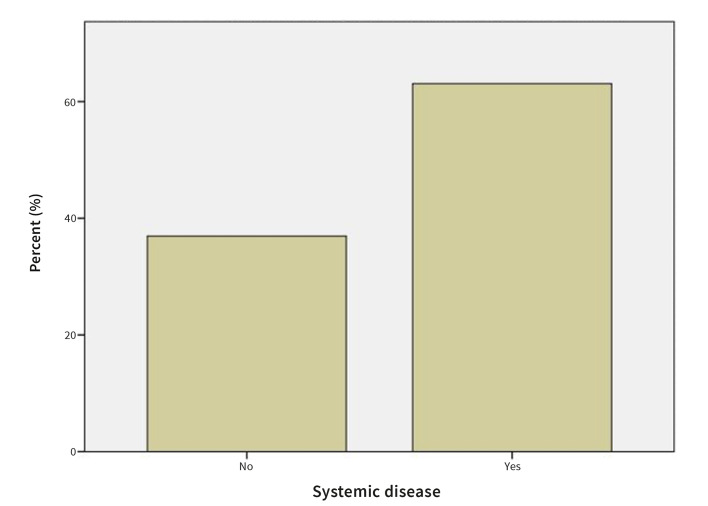
Prevalence of systemic diseases in OLP patients.

63.1% of the patients with OLP reported having one or more systemic diseases. Thyroid problems were reported in 22.5%, hypertension in 21.6%, hypercholesterolemia in 15.3%, gastrointestinal problems in 8.1%, cardiac problems in 8.1%, diabetes mellitus in 7.2 %, respiratory diseases in 6.3%, mental disorders in 5.4%, and osteoporosis in 3.6%. 2.7% of the patients reported having overcome cancer with a different combination of treatments, 1.8% reported having different skin diseases, and 0.9% reported having other systemic diseases (Fig 7).

#### Medication use

**Fig 8:**
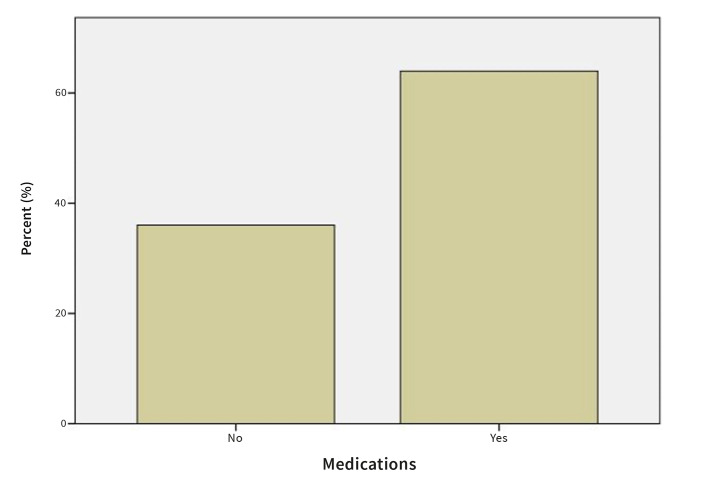
Prevalence of OLP patients taking medication.

64.0% of patients reported taking one or more medications. 22.5% reported taking antihypertensives, 19.8%, antithyroid medications, 14.4% statins, 9.9% medications for gastrointestinal problems, 7.2% antidiabetics, 4.5% antidepressants, 2.7% bisphosphonates and 0.9% other medications (Fig 8).

#### Smoking habits, number and type of cigarettes

**Fig 9:**
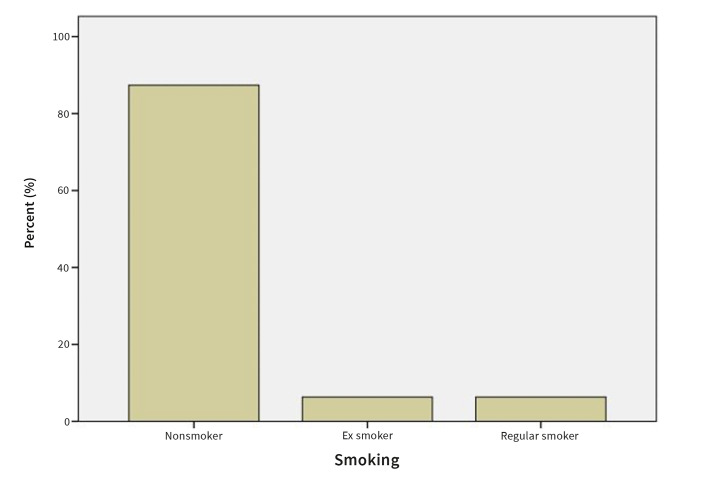
Prevalence of smoking in OLP patients.

**Fig 10:**
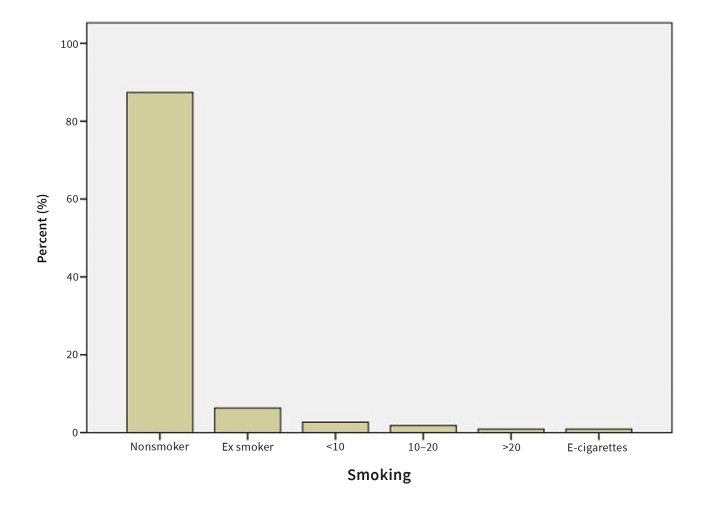
Prevalence of smoking in OLP patients by number and type of cigarettes.

87.0% of the patients reported being non-smokers, 6.3% reported being ex-smokers and 6.3% reported being regular smokers (Fig 9). 2.7% of the patients reported smoking <10 cigarettes per day, 1.8% reported smoking 10-20 cigarettes per day, and 0.9% patients reported ˃20 cigarettes per day. 0.9% patients reported smoking E-cigarettes (Fig 10).

#### Symptoms

**Fig 11:**
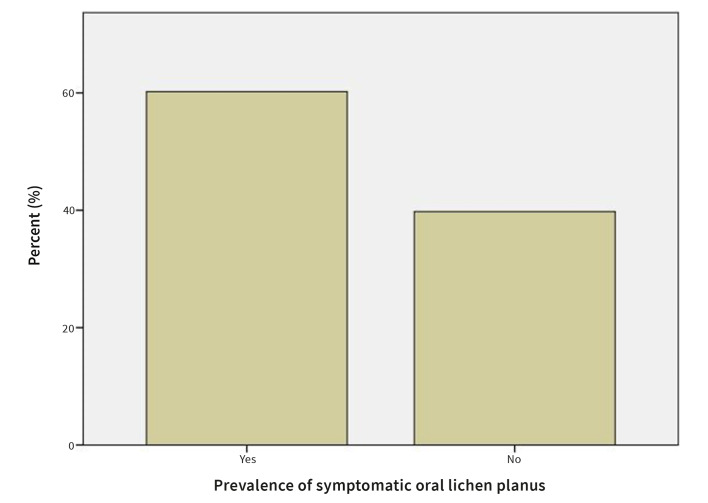
Prevalence of symptomatic OLP.

60.0% patients reported having symptoms, while 40.0% were asymptomatic. Symptoms reported were oral discomfort, soreness, or mucosal roughness (Fig 11).

#### Confirmation of OLP by histopathology or direct immunofluorescence assay

**Fig 12:**
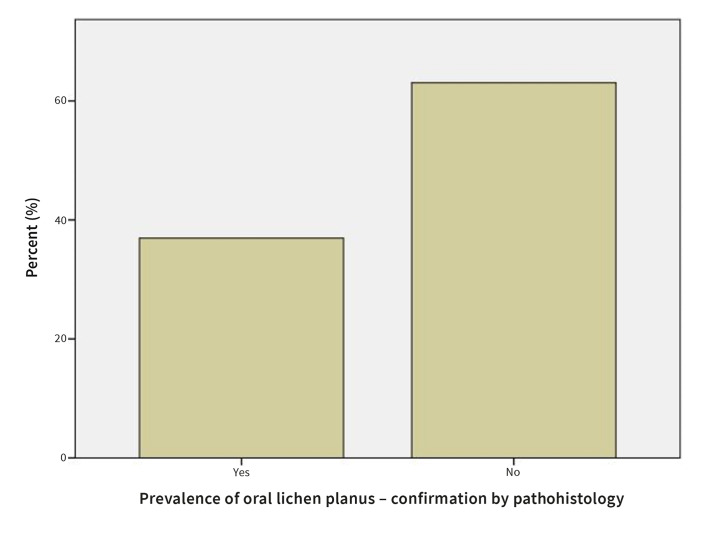
Prevalence of OLP confirmed by histopathology.

**Fig 13:**
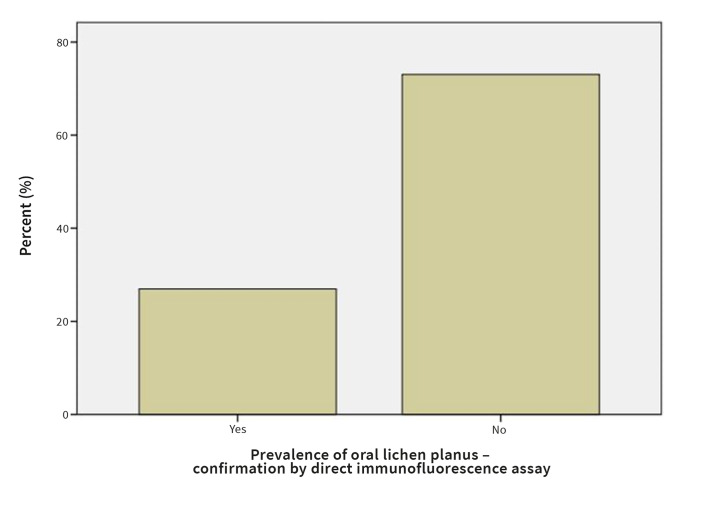
Prevalence of OLP confirmed by direct immunofluorescence.

In 36.0% of the patients diagnosis of OLP was confirmed histopathologically (Fig 12). In 29.7% patients, the diagnosis of OLP was confirmed by direct immunofluorescence assay (Fig 13).

## DISCUSSION

OLP is a common idiopathic disease affecting the oral mucosa by T-cell—mediated chronic inflammation.^8^ This study aimed to investigate the prevalence of OLP, its clinical features, and other associated factors in patients referred to the Department of Oral Diseases and Periodontology in Ljubljana, Slovenia, in the two-year period from 2022 to 2023. Out of the 1288 referred patients, 111 (8.6%) were diagnosed with OLP. The prevalence was higher than that reported worldwide, as the sample comprised patients who were referred to our Department due to problems in their oral cavity.

The characteristics of the patients included were similar to those reported in the literature, as described in the following.

79.3 % of patients in this study were women, which is in accordance with many other studies reporting the predominance of OLP in women.^25,27,37^ Gonzales Moles et al,^12^ however, failed to find a predominance of women among OLP patients.

The prevalence of OLP increased with age, as also reported in the systematic review and meta-analysis by Gonzales Moles et al.^12^ A predominance of OLP in patients aged >45 years was found, similar to results reported by Mostafa and Ahmed^25^ in a study on an Egyptian population.

The most commonly affected site in our sample was the buccal mucosa (44.1%), followed by the gingiva, tongue, and lips. In 51.4% of the patients, multiple sites were involved. The buccal mucosa was the most commonly affected site also in other studies.^3,38^ 54.1% of the patients presented with only reticular lesions, 4.5% with atrophic, and 1.8% with ulcerative OLP. 39.6% of patients were found to have multiple types of OLP in their oral cavity. Reticular lesions were found to be the most prevalent also in the studies by Persić et al,^31^ Thongprasom et al,^36^ and Pakfetrat et al.^30^ These results are not in accordance with the results obtained by Mostafa and Ahmed,^25^ who found the red type of OLP (atrophic and erosive lesions) to be most common.

5.4% of patients in our study had skin lesions. This is similar to the prevalence of OLP patients with skin lesions found in a study by Xue et al,^44^ less than Eisen^9^ as well as Carrozzo and Thorpe^4^ reported in their studies, and much less than that observed by Mostafa and Ahmed^25^ (23.44% of their subjects had skin lesions).

A few studies have investigated the periodontal status of patients with OLP. A review of these studies indicates that the periodontal state of OLP patients was significantly worse than in the control group.^1,23,33^ In our study, 13.5% of OLP patients presented with periodontal disease.

63.1% of the patients with OLP reported having one or more systemic diseases. Thyroid problems were reported in 22.5%, hypertension in 21.6%, hypercholesterolemia in 15.3%, gastrointestinal problems in 8.1%, cardiac problems in 8.1%, diabetes mellitus in 7.2%, respiratory diseases in 6.3%, mental disorders in 5.4%, and osteoporosis in 3.6%. 2.7% patients reported having overcome cancer with different combination of treatments, 1.8% reported having different skin diseases and 0.9% reported having other systemic diseases. These results are partially in accordance with the study by López-Jornet et al,^24^ which assessed 130 patients with OLP and found 19.2% of hypertension and 11.5% of diabetes mellitus in OLP patients. They did however find less hypercholesterolemia (11.5% vs 15.3%) and much less hypothyroidism (1.5% vs 22.5%).

Sixty-four percent (64%) of the patients with OLP reported taking one or more medications and 22.5% reported taking antihypertensives, which is in accordance with the results obtained by Gϋmrϋ, who reported that 23.8% of the OLP patients were taking antihypertensives.^15^ However, in their study, more patients reported taking antidiabetics (9.7% vs 7.2%) and antidepressives (8.4% vs 4.5%) and fewer patients reported taking antithyroid medications (4.9% vs 19.8%) and statins (7.0% vs 14.4%).

87.0% of the OLP patients reported being non-smokers, 6.3% ex-smokers, and 6.3% regular smokers. 2.7% reported smoking <10 cigarettes per day, 1.8% reported 10-20 cigarettes per day, and 0.9% patient reported smoking >20 cigarettes per day. These results agree with the study by Gorsky et al,^14^ which concluded that 61.5% of 187 assessed OLP patients were non-smokers, 22.5% were former smokers, and 16.0% were smokers.

The correlation between smoking and OLP is still not completely understood. Neumann-Jensen et al^26^ stated that OLP was more common in non-smoking patients than in smokers, which is in accordance with the present findings. Other authors, however, reported OLP being more common in smokers in a study among 7639 Indian villagers in Kerala, South India.^32^

60.0% of patients in our study reported having symptoms, while 40.0% were asymptomatic. Symptoms reported were oral discomfort, soreness ,or mucosal roughness. These results are very similar to the those obtained by Osipoff et al,^29^ where 61.0% of the OLP patients were classified as symptomatic.

Diagnosis of OLP relies on clinical examination, histopathology, and direct immunofluorescence. A total of 111 patients with a clinical or histopathological diagnosis of OLP that were referred to the Department of Oral Diseases and Periodontology, University Medical Center, Ljubljana, Slovenia were included in our study. In all 111 cases, a clinical diagnosis was suspected, but only symptomatic, atrophic, and ulcerative cases were also assessed histopathologically and by direct immunofluorescence assay. In 36.0% of the patients, the diagnosis of OLP was confirmed by histopathology, and in 29.7% patients the diagnosis of OLP was confirmed by the direct immunofluorescence assay.

Histopathological features of OLP include hyperparakeratosis, hyperorthokeratosis, Civatte bodies, basal cell hydropic change, and a band-like subepithelial lymphocytic infiltrate in the lamina propria. Additional findings are sawtooth rete ridges, atrophy, and acanthosis. Direct immunofluorescence assay can help support a diagnosis of OLP. This assay is especially important to differentiate OLP from other autoimmune diseases. The features of OLP found by direct immunofluorescence assay include deposition of fibrinogen in a shaggy pattern along the basement membrane in the absence of immunoglobulin (except for Civatte bodies coated by immunoglobulin) and no complement deposition.^5^

Our study is one of the few in Slovenia to report on the prevalence and related factors of OLP in patients referred to the Department of Oral Diseases and Periodontology in Ljubljana. It revealed the high OLP prevalence of 8.6%. However, other similar studies with precise diagnostic protocols are needed to confirm these findings.

## CONCLUSION

The prevalence of oral lichen planus in patients referred to the Department of Oral Diseases and Periodontology in Ljubljana, Slovenia, is high. These findings highlight the importance of careful oral mucosa examination in routine clinical practice. Dentists should be particularly attentive in older patients, especially women, and consider appropriate diagnostic confirmation in clinically unclear cases.

## ACKNOWLEDGEMENTS

This study was supported by a grant from Slovenian Research Agency, Ljubljana, Slovenia (No. P3–0293).
